# Yes, We Have an Inflation of Reviews: But of the Wrong Kind!

**DOI:** 10.3389/fpls.2016.00494

**Published:** 2016-04-26

**Authors:** David G. Robinson

**Affiliations:** Department of Plant Cell Biology, Centre for Organismal Studies, University of HeidelbergHeidelberg, Germany

**Keywords:** scientific writing, review articles, catalog reviews, critical reviews, open debates

I read with interest Ingo Schubert's short article on the inflationary number and value of review papers (*Front. Plant Sci. 7:88. doi: 10.3389/fpls.2016.00088)*. I wholeheartedly agree with Schubert that far too many reviews are getting published. Many are what I call “catalog” reviews, and any good Ph.D student worth his salt can, with the help of PubMed, make a compilation of the recent (last 5 years) literature on a particular topic within days. Reviews of this kind seldom draw attention to areas of conflict, rarely provide insight into technical difficulties, and often fail to point to challenges for future research.

Publishing reviews is an established strategy through which journals can increase their IFs and thereby enlarging the circle of readership. The irony is that the reviews published by the increasing number of third rate journals are of the catalog type and therefore don't get cited. In agreement with Ingo Schubert I would therefore appeal to younger scientists not to succumb to the temptation of writing this kind of review for that class of journal. They are of questionable value and hardly boost one's reputation.

Believe that critical reviews are the only type of review article that should be asked for and be delivered. What are the prequisites for the preparation of a critical review? The authors need to be accredited leaders in the field (unfortunately, this is by no means always the case), they should make the effort to read and evaluate the original papers they cite rather than simply cite what was previously cited by others. Above all, they need to accomodate the reader's desire to be directed to controversies, but also to take a standpoint on them. These may seem banal criteria, but they remain frequently unfulfilled. The essence of a good review is not so much as to highlight individual papers but to critically evaluate all the relevant papers on a particular subject.

Would like to draw attention to an alternative to a high profile review to focus interest on controversial topical issues. Essentially this is an open debate with the conflicting parties putting their case in writing for the scientific public to judge for themselves whose arguments are the more convincing. I have tried this out twice, in both cases with an extremely positive feedback from reviewers/editors. My first attempt, over 20 years ago, was to put the spotlight on the auxin binding protein (ABP; Hertel, 1995; Venis, 1995). At that time I had become involved in ABP research by being asked by Mike Venis to apply a silver-immmunogold enhanced epipolarzation technique to visualize ABP at the surface of maize coleoptile protoplasts (Diekmann et al., 1995). From the referees reports to this paper I realized just how volatile the situation with ABP was. Since I also knew one of the major opponents to ABP research (Rainer Hertel) quite well, I thought it would be a rewarding experience for the plant community to have the two major opponents in the ABP field engage in an intellectual duel. Not being an ABP-researcher myself it is difficult for me in retrospect to judge how effective this exercise in scientific pugilism was in generating more interest, but it is interesting to note that, after many years of relative stagnation, interest in ABP research has recently flared up (Grones and Friml, 2015).

More recently, I managed to persuade three experienced colleagues to voice their opinions on vesicles vs. tubes as a means of transport between the endoplasmic reticulum and the Golgi apparatus (Robinson et al., 2015). This controversy had been going on for years with a number of one-sided reviews in the literature. Again I thought it was time for a public debate on this issue. Based on the comments that I have received, this is undoubtedly the best way to focus attention on a hot topic. But, in the sense that it reveals how established researchers approach and try to solve problems, such articles are of immeasurable didactical value to young scientists. To get such ventures going requires that senior scientists in a particular area take the initiative and volunteer to act as coordinators. This not only involves persuading competitors to “put on the gloves,” but, as I did for the vesicles vs. tubules “fight” to be prepared to set the stage by writing an introduction which draws attention to the conflict under consideration. Ideally, and to promulgate further discussion, journals should also open a discussion forum in a following issue for comments. I encourage others to follow my example and explore further this kind of scientific debate as a worthwhile alternative to conventional reviews.

## Author contributions

The author confirms being the sole contributor of this work and approved it for publication.

### Conflict of interest statement

The author declares that the research was conducted in the absence of any commercial or financial relationships that could be construed as a potential conflict of interest.
